# Positive Feedback of *NDT80* Expression Ensures Irreversible Meiotic Commitment in Budding Yeast

**DOI:** 10.1371/journal.pgen.1004398

**Published:** 2014-06-05

**Authors:** Dai Tsuchiya, Yang Yang, Soni Lacefield

**Affiliations:** Department of Biology, Indiana University, Bloomington, Indiana, United States of America; National Cancer Institute, United States of America

## Abstract

In budding yeast, meiotic commitment is the irreversible continuation of the developmental path of meiosis. After reaching meiotic commitment, cells finish meiosis and gametogenesis, even in the absence of the meiosis-inducing signal. In contrast, if the meiosis-inducing signal is removed and the mitosis-inducing signal is provided prior to reaching meiotic commitment, cells exit meiosis and return to mitosis. Previous work has shown that cells commit to meiosis after prophase I but before entering the meiotic divisions. Since the Ndt80 transcription factor induces expression of middle meiosis genes necessary for the meiotic divisions, we examined the role of the *NDT80* transcriptional network in meiotic commitment. Using a microfluidic approach to analyze single cells, we found that cells commit to meiosis in prometaphase I, after the induction of the Ndt80-dependent genes. Our results showed that high-level expression of *NDT80* is important for the timing and irreversibility of meiotic commitment. A modest reduction in *NDT80* levels delayed meiotic commitment based on meiotic stages, although the timing of each meiotic stage was similar to that of wildtype cells. A further reduction of *NDT80* resulted in the surprising finding of inappropriately uncommitted cells: withdrawal of the meiosis-inducing signal and addition of the mitosis-inducing signal to cells at stages beyond metaphase I caused return to mitosis, leading to multi-nucleate cells. Since Ndt80 enhances its own transcription through positive feedback, we tested whether positive feedback ensured the irreversibility of meiotic commitment. Ablating positive feedback in *NDT80* expression resulted in a complete loss of meiotic commitment. These findings suggest that irreversibility of meiotic commitment is a consequence of the *NDT80* transcriptional positive feedback loop, which provides the high-level of Ndt80 required for the developmental switch of meiotic commitment. These results also illustrate the importance of irreversible meiotic commitment for maintaining genome integrity by preventing formation of multi-nucleate cells.

## Author Summary

There are two main types of cell division cycles, mitosis and meiosis. During mitosis, DNA is replicated and then chromosomes segregate, producing two daughter cells with the same ploidy as the progenitor cell. During meiosis, DNA is replicated and then chromosomes undergo two rounds of segregation, producing four gametes with half the ploidy of the progenitor cell. As the cell enters into the meiotic divisions, it irreversibly commits to finishing meiosis and cannot return to mitosis. The molecular mechanisms that define meiotic commitment are not well understood. In this study, we asked whether the regulatory network involved in the transcription of *NDT80* has a role in meiotic commitment. Ndt80 is a transcription factor that induces the genes needed for the meiotic divisions. We found that a high-level of Ndt80 activity is required for meiotic commitment; in wildtype cells, this is achieved through a transcriptional positive feedback loop– a regulatory mechanism in which the Ndt80 protein increases the transcription of its own gene. In the absence of positive feedback, cells escape meiosis inappropriately, resulting in an aberrant cell cycle that causes an increase in genome copy number. This study shows the important role of positive feedback in meiotic commitment and in the maintenance of genome integrity.

## Introduction

During gametogenesis, cells integrate external signals with internal cell-cycle control mechanisms to initiate and sustain meiosis, and eventually to differentiate into gametes. Although the external signals that initiate the switch into meiosis in various organisms are quite diverse, many of the features of meiosis are universal in the production of haploid meiotic products from a diploid progenitor cell [1], [2]. After cells enter into meiosis, maintenance of meiosis is important to ensure proper gametogenesis. In humans, an inability to properly maintain meiosis can result in developmental abnormalities or possibly oncogenesis in the germ line [1].

In many organisms, cells that have initiated meiosis pass through an irreversible transition near the end of prophase I. These cells irreversibly commit to undergoing the meiotic divisions. Cells in prophase I have undergone pre-meiotic DNA replication and have initiated meiosis-specific events such as double strand break (DSB) formation, pairing of homologous chromosomes, synaptonemal complex (SC) assembly, and the initiation of recombination, but they have not yet entered into the meiotic divisions [2]. Indeed, human, mouse, and frog oocytes arrest at the end of prophase I and enter into the meiotic divisions only if stimulated by hormones [3], [4]. The hormones induce resumption of meiosis and the oocyte becomes committed to finishing meiosis I. In the *D. melanogaster* ovarian cyst, 16 cells enter into meiosis; however, at prophase I, only 1 cell, chosen as the oocyte, continues meiosis [5]–[8]. The other 15 cells will exit meiosis and enter an endocycle. In *S. cerevisiae*, cells commit to meiosis as they exit prophase I and enter the meiotic divisions [9]. In a process termed return-to-growth (RTG), budding yeast cells in prophase I exit meiosis and return to mitosis if the meiosis-inducing signal is withdrawn and the mitosis-inducing signal is provided [10]–[14]. Once they have passed the commitment point, cells are committed to meiosis even without the continued presence of the meiosis-inducing signal. An understanding of the regulatory mechanisms that drive cells through meiotic commitment points will provide insight into mechanisms that constrain cells to a developmental path.

The ability of budding yeast cells to make the developmental switch from meiosis back to mitosis confers upon them the advantage to alter their developmental program in response to fluctuating environmental conditions [9]. Nutrient limitation induces the process of sporulation, in which cells enter meiosis and then package the meiotic products into spores. The spores can survive adverse conditions and then germinate when nutrients become available. If nutrient-rich conditions return prior to cells reaching the meiotic commitment point, cells exit meiosis and return to mitosis.

In budding yeast, the temporal coordination of cell-cycle events in meiosis is tightly controlled and intertwined with transcriptional cascades [15], [16]. Cells initiate meiosis when starved of nitrogen and glucose but provided acetate as an energy source. The starvation signal stimulates the expression of the Ime1 transcription factor, which induces a class of early genes required for the entry into meiosis, pre-meiotic DNA replication, and prophase I. At pachytene of prophase I, the paired chromosomes have formed extensive synaptonemal complex and have initiated crossing-over. To exit pachytene of prophase I, the Ndt80 transcription factor is induced and turns on ∼150 middle meiosis genes by binding to a midsporulation element (MSE) in the promoter of the genes [17]–[20]. Once the Ndt80-dependent genes are expressed, the SC disassembles, a meiotic spindle forms, and cells segregate homologous centromeres in meiosis I and sister centromeres in meiosis II [16], [21]. As cells are undergoing meiosis II, a wave of late genes is expressed, many of which encode proteins required for the packaging of the four meiotic products into spores [15].

Here, we investigated the irreversibility of meiotic commitment. We hypothesized that the *NDT80* transcriptional regulatory network was essential for generating irreversibility due to its requirement in meiotic commitment, its sensitivity to nutritional changes, and its activation through positive feedback to give a high-level burst of *NDT80* expression [16], [17], [21], [22]. The Ime1 transcription factor induces *NDT80* transcription by binding an upstream regulatory sequence within the promoter [17]. *NDT80* is expressed later than most Ime1-dependent genes because the *NDT80* promoter is regulated by a repressor complex comprised of Sum1, Rfm1, and a histone deacetylase Hst1 [23], [24]. The loss of repression occurs after the phosphorylation of Sum1 by multiple kinases at the end of pachytene [25]–[27]. A high-level of *NDT80* expression is induced by an autoregulatory positive feedback loop in which Ndt80 binds to MSEs in its own promoter and enhances its own transcription [16], [17]. Since positive feedback loops are often found in irreversible cell-cycle transitions [28], the *NDT80* transcriptional network becomes a strong candidate for generating the irreversibility of meiotic commitment.

Using single-cell analysis, we analyzed the role of the *NDT80* transcriptional network in regulating the irreversibility of meiotic commitment. We found that cells commit to meiosis in prometaphase I, after the induction of the Ndt80-dependent genes. And, that high-level induction of *NDT80* was needed for the irreversibility of meiotic commitment. By making strains that allowed us to manipulate both the timing and level of *NDT80* expression, we showed that decreasing the levels of *NDT80* could uncouple the entrance into meiosis and meiotic commitment. We found cells that were inappropriately uncommitted to meiosis; these cells underwent meiosis I and then returned to mitosis instead of finishing meiosis II, becoming multi-nucleate polyploid cells. Further reducing the levels of *NDT80* by making *NDT80* promoter mutations to disrupt positive feedback resulted in a complete loss of meiotic commitment. With complete medium addition, all of the cells returned to mitosis from stages beyond metaphase I. Our work suggests that a threshold level of Ndt80 is needed for the irreversibility of meiotic commitment and that positive feedback in the *NDT80* transcriptional regulatory network ensures that threshold level.

## Results

### A microfluidics assay to determine the cell-cycle timing of meiotic commitment in individual cells

**Figure 1:**
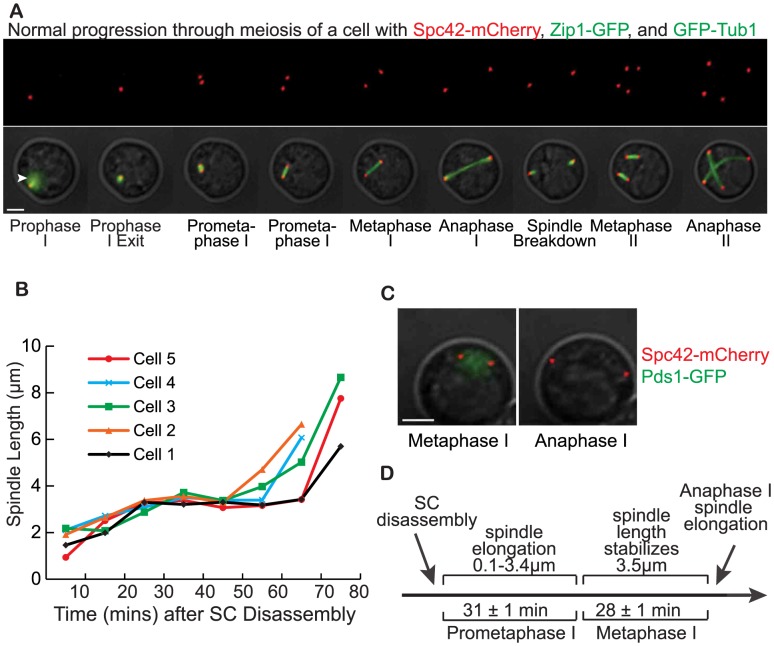
Defining the stages of meiosis with time-lapse microscopy. A) Cell with Spc42-mCherry, Zip1-GFP, and GFP-TUB1 were imaged using time-lapse microscopy. Still pictures from the time-lapse fluorescence microscopy representing the different stages of meiosis are shown. Fluorescent images are overlaid with a DIC image (Scale bar - 3 µm). The white arrowhead marks the SC, shown with Zip1-GFP. B) A plot of spindle length in µm (Y-axis) at 10-minute timepoints after SC disassembly (X-axis) of 5 cells undergoing meiosis. The distances between SPBs were measured to obtain the spindle length as the cells progressed from prophase I to anaphase I. C) Cell with Pds1-GFP and Spc42-mCherry shows the spindle length at the metaphase I to anaphase I transition when Pds1-GFP is degraded (Scale bar - 3 µm). D) Diagram depicting the timing of the stages of prometaphase I and metaphase I based on spindle length measurements.

Past studies performed on populations of cells and one study on individual cells showed that meiotic commitment occurs after pachytene, but before the first meiotic division [9], [29]. The meiotic commitment point was thought to occur at the end of prophase I [10], [16], and predicted to be associated with the initiation of the separation of spindle pole bodies (SPBs), the yeast equivalent of the centrosome [9], [30]. We wanted to define the stage of meiotic commitment more precisely and needed to establish markers to differentiate prophase I exit, prometaphase I, metaphase I, and anaphase I. As cells exit prophase I, the transcription factor Ndt80 induces the transcription of the middle meiosis genes, including the M-phase cyclins, which are needed for spindle assembly and the meiotic divisions [15], [21], [31], [32]. As the bipolar spindle assembles, the cells are transitioning from prometaphase I to metaphase I. In anaphase I, the spindle elongates. Therefore, we decided to use the changes in spindle length to determine the meiotic stages. With time-lapse microscopy, we captured images every 10 minutes as cells underwent meiosis. We followed the expression of three proteins tagged with fluorescent markers: Spc42-mCherry, Zip1-GFP, and GFP-Tub1 (Figure 1A). Spc42, a component of the spindle pole body (SPB), the yeast equivalent of the centrosome, was fused to mCherry, allowing us to monitor the separation of the SPBs, and therefore the length of the spindle [33]. Zip1, a component of the SC, marks pachytene [34], [35]. The disassembly of the SC and concomitant loss of Zip1-GFP localization represents the end of prophase I. GFP-Tub1, which encodes α-tubulin fused to GFP, allowed us to monitor different meiotic stages based on spindle morphology [36], [37]. Although Zip1 and Tub1 are both fused to GFP, the locations of the proteins are morphologically distinct [37], [38] (Figure 1A). To ensure that we could indeed differentiate between Zip1-GFP and GFP-Tub1, we measured the time from SC disassembly to SPB separation in cells with Zip1-GFP and Spc42-mCherry and compared this time to cells with all three marked proteins. In both strains, we see that the SPBs separate approximately 3 minutes after SC loss (n = 100 cells per genotype, SI Figure S1A, B), suggesting that we can differentiate between Zip1-GFP and GFP-Tub1.

To more precisely define prometaphase I and metaphase I, we measured the distance between SPBs to determine spindle lengths as cells progressed from prophase I to anaphase I. The analysis of the spindle length showed that after the SC disassembles, the spindle undergoes a period of elongation for 31±1 mins (average time ± S.E., n = 75 cells) (Table 1). Once the spindle reaches a length of 3.5±0.05 µm, the spindle maintains that length for 28±1 mins. The spindle will increase its length to 4.4±0.07 µm and then elongates further and chromosomes segregate in anaphase I. In Figure 1B, we plotted the spindle length at timepoints between the end of prophase I and the beginning of anaphase I for 5 of the 75 cells that we analyzed. We defined prometaphase I as the time after SC disassembly in which the SPBs are separating and the spindle is elongating from 0.1–3.4 µm. We defined the start of metaphase I as the time in which the spindle reaches the stable length of 3.5 µm. To determine whether the spindle elongation from to 3.5 µm to 4.4 µm marked the transition into anaphase I, we used a strain with Spc42-mCherry and GFP-tagged securin (Pds1 in budding yeast). Since Pds1 is degraded at the metaphase I to anaphase I transition, we monitored the spindle length at the timepoint just prior to Pds1-GFP degradation to define the spindle length at the end of metaphase I (Figure 1C). We found that the spindle was on average 3.5±0.05 µm at the last timepoint the cells were in metaphase I (n = 70 cells, average spindle length ± S.E.). In the first timepoint after Pds1 degradation, the cells enter anaphase I and the spindle lengths ranged from 3.8 to 9.1 µm. Therefore, we defined metaphase I as the time in which the spindle has reached the length of 3.5 µm and anaphase I at the time in which the spindle elongates beyond 3.5 µm (Figure 1D). We next used these defined meiotic stages to pinpoint meiotic commitment.

**Table 1:** The duration of the meiotic stages (time in minutes ± standard error. n = 75 cells analyzed for wildtype and 80 cells analyzed for *NDT80/ndt80*Δ).

Genotype	Prometaphase I	Metaphase I	Anaphase I	Metaphase II	Anaphase II
wildtype	31±1	28±1	17±0.6	37±1	90±1
*NDT80/ndt80*Δ	28±1	21±1	16±0.6	31±1.2	93±2

**Figure 2:**
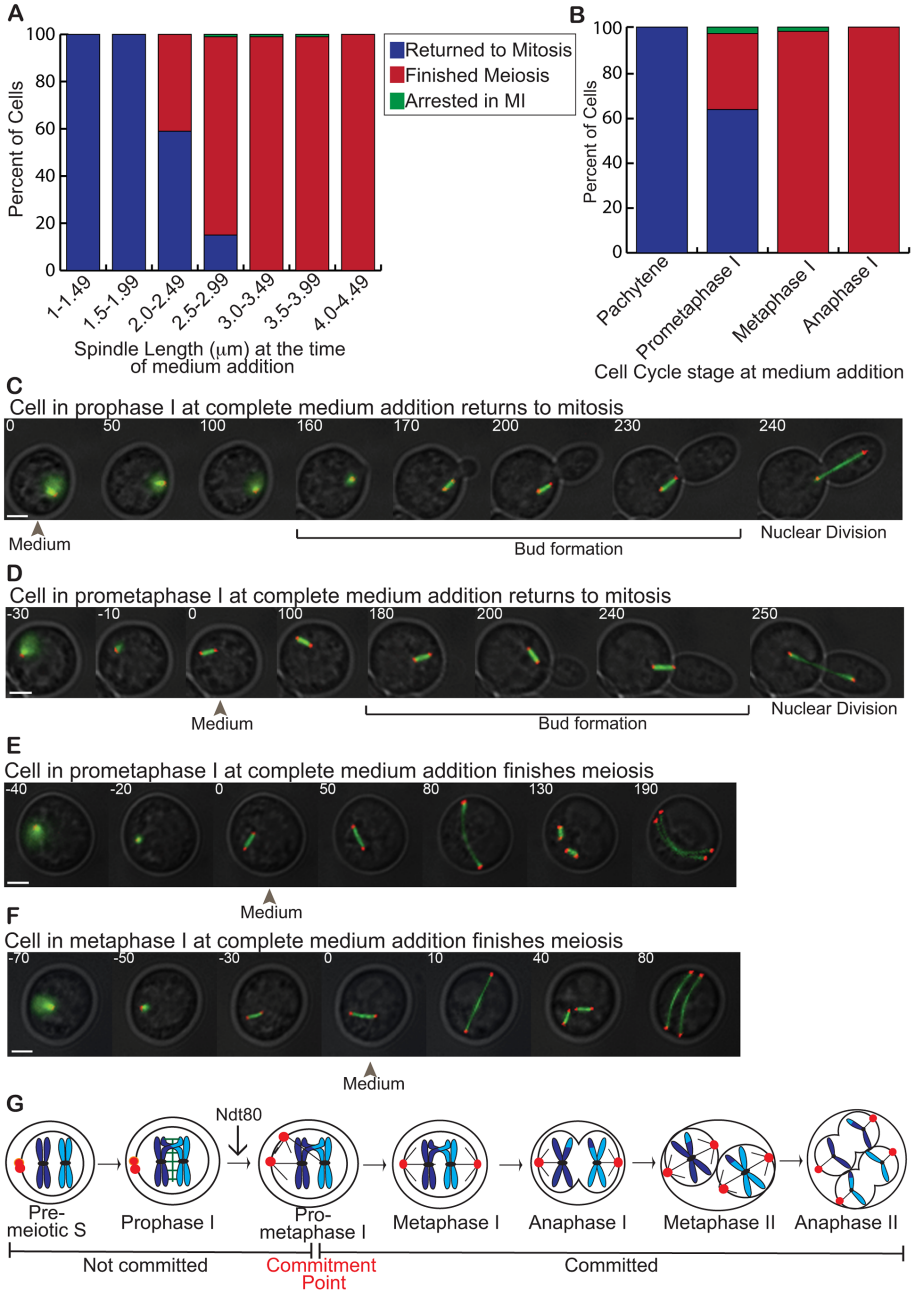
The meiotic commitment point occurs in prometaphase I. A) Graph of the percent of cells with each cell-cycle outcome (y-axis) upon complete medium addition to cells with different spindle lengths (x-axis). Cells that returned to mitosis are shown in blue, finished meiosis are shown in red, and arrested in meiosis I are shown in green. B) Graph of the percent of cells with each cell-cycle outcome (y-axis) at the meiotic stage in which complete medium was added. Cells that returned to mitosis are shown in blue, finished meiosis are shown in red, and arrested in meiosis I are shown in green. C-F) Cells with Spc42-mCherry, Zip1-GFP, and GFP-TUB1 initiated meiosis in microfluidic chambers. Complete medium was flowed into the chambers (at timepoint with grey arrowhead) and time-lapse microscopy was used to monitor the cell-cycle outcome. Fluorescent images are overlaid with a DIC image (Scale bar – 3 µm). C) Cell in prophase I at the time of complete medium addition returned to mitosis. D) Cell in prometaphase I at the time of complete medium addition returned to mitosis. E) Cell in prometaphase I at the time of complete medium addition finished meiosis. F) Cell in metaphase I at the time of complete medium addition finished meiosis. G) Diagram of the meiotic commitment point. SPBs are shown in red, chromosomes in blue, SC in green, and spindle microtubules in grey. Cells commit to meiosis as the SPBs are separating in prometaphase I.

To monitor meiotic commitment, we used a microfluidics assay coupled to time-lapse microscopy to monitor individual cells [38]. W303 cells were placed in microfluidic chambers and introduced to sporulation medium to induce meiosis. After 12 hours, cells were at a variety of meiotic stages, and synthetic complete medium was flowed into the chambers. Cells were scored based on their spindle length when exposed to complete medium and their cell-cycle outcomes: returned to mitosis, finished meiosis, or arrested. We found that as the spindle length increased, the percent of cells committed to meiosis also increased (n = 300, Figure 2A, Sup. Table S2). If complete medium was added to cells with a spindle length between 1.0–1.99 µm, the cells returned to mitosis. If complete medium was added to cells with a spindle length from 3.0–5.5 µm, the cells finished meiosis. In cells with spindle lengths between 2.0–2.99 µm, some cells returned to mitosis and some cells finished meiosis. Since spindle assembly requires the activity of the M-phase cyclins whose transcription is dependent on Ndt80, our findings suggest that cells commit to meiosis after the expression of the Ndt80-dependent genes.

We next assigned the cells a meiotic stage based on their spindle length (Figure 2B), and found that 100% of cells in prophase I returned to mitosis upon complete medium addition, as previously reported (n = 100, Figure 2B,C, Supp. Table S3) [9]. Of cells in prometaphase I upon complete medium addition, 64% returned to mitosis (Video S1, Figure 2B,D), 34% finished meiosis (Video S2, Figure 2B,E), and 2% arrested, in meiosis I (n = 100). Finally, 99% of cells finished meiosis upon complete medium addition in metaphase I (n = 100, Figure 2B,F). These data show that the meiotic commitment point lies in prometaphase I (Figure 2G). Prior experiments did not have the sensitivity to resolve these variations in cell-cycle outcomes upon nutrient addition in prometaphase I [9].

**Figure 3:**
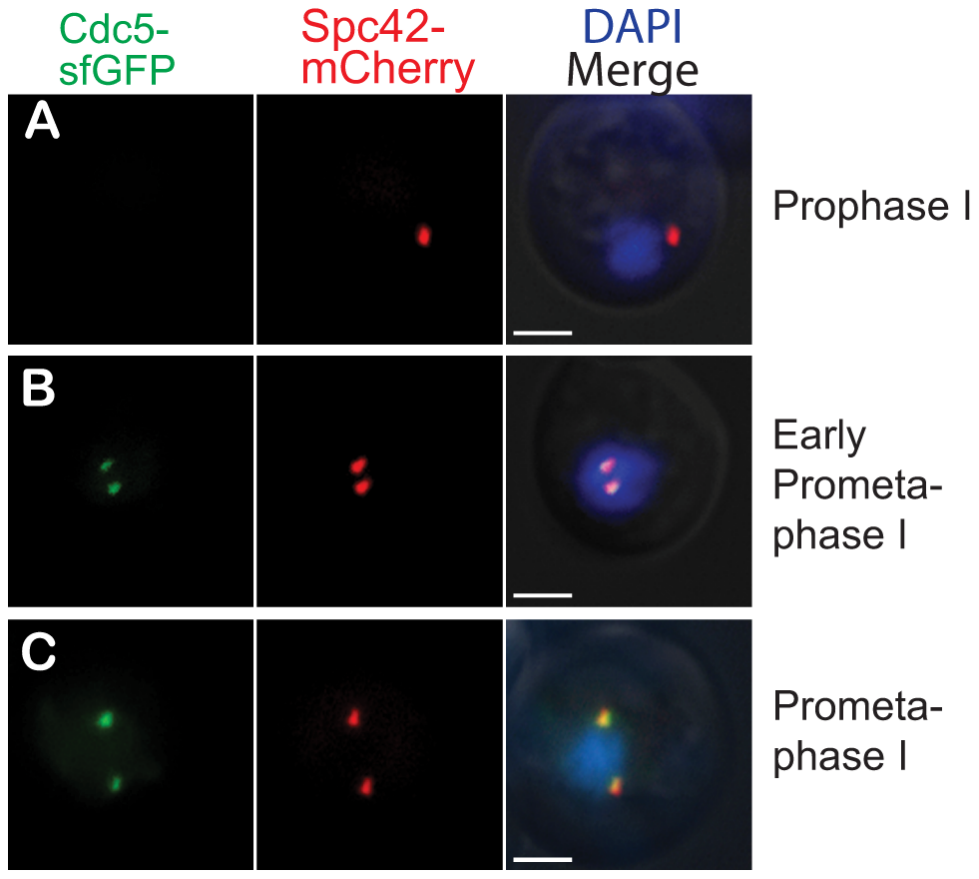
Cdc5-sfGFP is present prior to meiotic commitment. Fluorescence microscopy of cells with Cdc5-sfGFP, Spc42-mCherry, and DAPI staining. Scale bar 2 µm. A) Cdc5-sfGFP is not present in prophase I. B) Cdc5-sfGFP is present in early prometaphase I, prior to the meiotic commitment point (spindle length of 0.73 µm). C) Cdc5-sfGFP is present in prometaphase I (spindle length of 2.38 µm).

Prometaphase I occurs after the synaptonemal complex is disassembled and after spindle formation initiates. This suggests that prior to commitment, Ndt80 has started transcribing the Ndt80-dependent genes such as the M phase cyclins, which are needed for spindle formation. To verify that the Ndt80-dependent genes were indeed transcribed and translated prior to commitment, we monitored the timing of the accumulation of the encoded protein of the Ndt80-dependent gene CDC5, which encodes polo kinase. Cdc5 is necessary and sufficient for SC disassembly at the end of prophase I [39]. To determine whether Cdc5 was present prior to commitment in prometaphase I, we tagged Cdc5 with Superfolder-GFP (Cdc5-sfGFP), a fast folding variant of GFP [40], [41] in a strain with Spc42-mCherry. As expected, cells in prophase I that have not yet expressed *NDT80* do not have Cdc5-sfGFP present (Figure 3A). In contrast, 100% of cells in prometaphase I, with a spindle length between 0.1–3.4 µm, have Cdc5-GFP present (n = 44 cells) (Figure 3B-C). These results demonstrate that the Ndt80-dependent gene CDC5 is expressed and the protein is present in the cell prior to meiotic commitment.

### Altered expression of *NDT80* results in inappropriately uncommitted cells

Although cells commit to meiosis after the initiation of the expression of the Ndt80-dependent genes, we considered that Ndt80 could have a role in the irreversibility of meiotic commitment due to its tightly regulated transcriptional activation. We asked if modifying *NDT80* expression could lead to an alteration in the establishment of meiotic commitment. We placed *NDT80* under the control of the *GAL1,10* promoter (*P_GAL1,10-_NDT80*) and the Gal4 activator was fused to the estradiol receptor so that the addition of estradiol activated *NDT80* expression via Gal4 (*GAL4-ER*) [32], [42]. The *P_GAL1,10-_NDT80/P_GAL1,10-_NDT80* cells undergo meiosis and form spores with the addition of estradiol, as previously described [32]. Because the *NDT80* promoter is absent in the *P_GAL1,10-_NDT80* cells, *NDT80* transcription is no longer subject to transcriptional positive feedback, but instead can be regulated by an inducible promoter to limit the duration of transcription.

**Figure 4:**
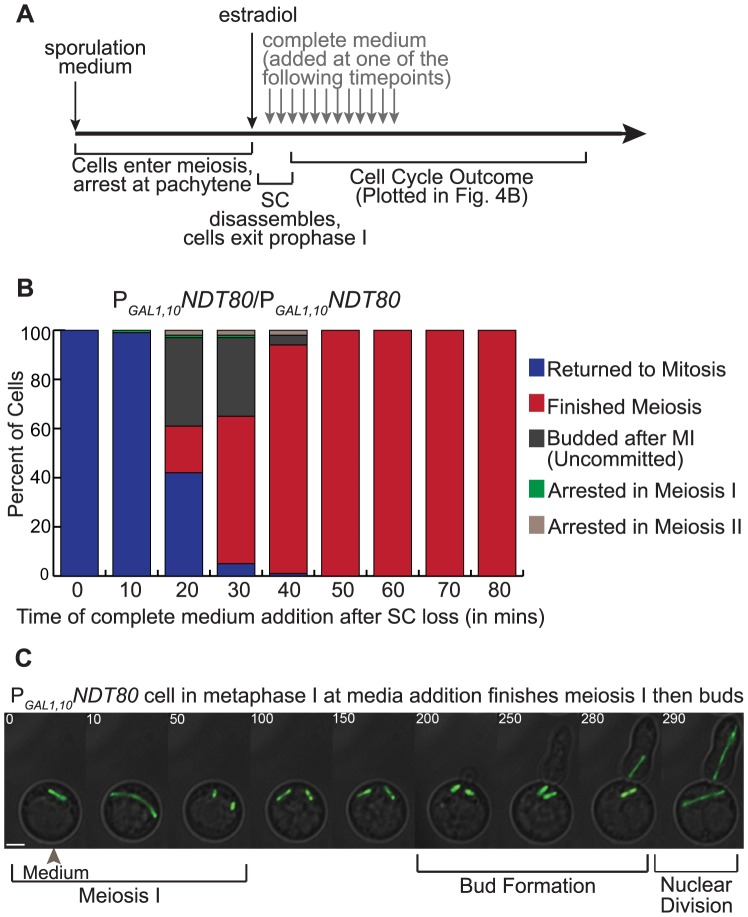
Altering *NDT80* expression results in inappropriately uncommitted cells. A) Diagram of the experimental method. Cells in microfluidic chambers are first exposed to sporulation medium. The cells enter meiosis and arrest at pachytene (due to the lack of *NDT80* expression). Beta-estradiol is added to induce *NDT80* and the cells exit prophase I. Complete medium is added to cells in different chambers at one of the 10-minute timepoints shown after the addition of estradiol. Cell Cycle outcomes are recorded. B) Graph of the percent of cells at each cell-cycle outcome (y-axis) upon complete medium addition at timepoints after SC disassembly (in minutes) (x-axis). Cells that returned to mitosis are in blue. Cells that finished meiosis are in red. Cells that were inappropriately uncommitted are in dark grey. Cells that arrested are in brown and green. C) Time-lapse images from a cell that is inappropriately uncommitted. The cell expresses GFP-Tub1. Complete medium was added to the cell in metaphase I (timepoint with grey arrowhead). The cell underwent meiosis I, assembled two spindles, budded and then underwent nuclear division, in which two nuclei divided (Scale bar - 3 µm).

*P_GAL1,10-_NDT80/P_GAL1,10-_NDT80* cells in microfluidics chambers were exposed to sporulation medium to induce meiosis until they arrested at pachytene (due to the lack of Ndt80). Estradiol was added to induce *NDT80*, and, at 10-minute timepoints after the induction of *NDT80*, we introduced complete medium into separate chambers and recorded cell-cycle outcomes of each chamber (Figure 4A). The addition of complete medium, which contains glucose but not estradiol, represses the transcription of the *GAL1,10* promoter. In Figure 4B, we have plotted the percent of cells for each cell-cycle outcome (y-axis) when complete medium was added at a timepoint after the cells disassembled the SC and exited prophase I (x-axis) (n = 100 cells counted for each timepoint)(Supp. Table S4). When complete medium was added ten minutes after SC loss, 99% of the cells returned to mitosis. However, if complete medium was added 30 minutes after SC loss, only 5% of cells returned to mitosis; 60% finished meiosis; 3% arrested; and, the remaining 32% of cells took another path, described below. By 50 minutes after SC loss, enough Ndt80 had accumulated such that 100% of the cells were committed to meiosis and finished meiosis after the addition of complete medium.

Importantly, we found an additional cell-cycle outcome in the inducible *NDT80* strain: those cells that completed meiosis I, assembled two spindles, then returned to mitosis as demonstrated by bud formation (Video S3, Figure 4B,C). After budding, both nuclei divided, resulting in multi-nucleate cells. We defined these cells as inappropriately uncommitted –they underwent meiosis I but were not committed to finishing meiosis II and instead returned to mitosis. This is a novel behavior representing the absence of commitment, and different from the cells that are not yet committed to meiosis I and returned to mitosis from prophase I. When complete medium was added 20 minutes and 30 minutes after SC loss, 36% and 32% of cells were inappropriately uncommitted, respectively. Also, at the 20- and 30-minute timepoints, the percentage of cells that returned to mitosis decreased and those that finished meiosis increased when compared to the 10-minute timepoint. These data suggest that, with an inducible *NDT80*, either the duration of *NDT80* transcription or the level of Ndt80 protein after SC breakdown is important for meiotic commitment. With *NDT80* expressed for a short time period (0–10 mins) after SC disassembly, the cells returned to mitosis. With *NDT80* expressed for a longer time period (50 minutes or more), the cells committed to meiosis. We propose that at timepoints in between (20–40 minutes), Ndt80 may be at intermediate levels: i) some cells had Ndt80 below a threshold level and returned to mitosis; ii) some cells had Ndt80 above a threshold level and committed to meiosis, and; iii) some cells had enough Ndt80 to finish meiosis I, but not enough Ndt80 to commit to meiosis II and returned to mitosis after meiosis I.

### Meiotic commitment requires a high-level of *NDT80* Expression

**Figure 5:**
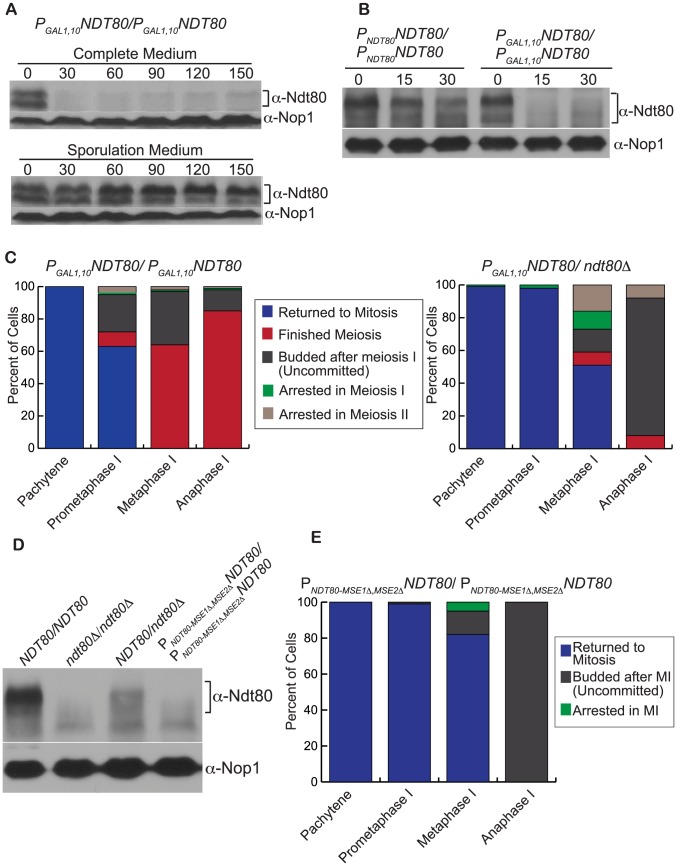
Decreasing the levels of Ndt80 results in fewer cells that commit to meiosis. A) Analysis of Ndt80 protein levels in *P_GAL1,10_NDT80/P_GAL1,10_NDT80* cells. Beta-estradiol was added to cells in prophase I to induce the expression of *NDT80*. After 110 minutes of Ndt80 expression (t = 0 minutes), complete medium without beta-estradiol (complete medium) or sporulation medium with beta-estradiol (sporulation medium) was added to the cells and protein was collected every 30 minutes for 150 minutes. Western blot was performed using anti-Ndt80 and anti-Nop1 (loading control) antibodies. B) Analysis of Ndt80 protein levels in *P_NDT80_NDT80/P_NDT80_NDT80* and *P_GAL1,10_NDT80/P_GAL1,10_NDT80* cells. Beta-estradiol was added to cells in prophase I to induce the expression of *NDT80*. After 110 minutes of Ndt80 expression, complete medium without beta-estradiol (complete medium) was added to the cells (t = 0 minutes) and protein was collected every 15 minutes for 30 minutes. Western blot was performed using anti-Ndt80 and anti-Nop1 (loading control) antibodies. C) Comparison plots of *P_GAL1,10_NDT80*/*P_GAL1,10_NDT80* and *P_GAL1,10_NDT80/ndt80Δ* strains. Graphs show the percent of cells with each cell cycle outcome (y-axis) upon nutrient addition at different meiotic stages (x-axis). Cells that returned to meiosis are in blue. Those that finish meiosis are in red. Cells that were inappropriately uncommitted are in dark grey. Cells that arrested are in brown or green. D) Analysis of Ndt80 protein levels in different strain backgrounds. Western blot was performed using anti-Ndt80 and anti-Nop1 (loading control) antibodies. E) Plot of the *P_NDT80-MSE1Δ,MSE2Δ_-NDT80* strain, in which positive feedback in the *NDT80* promoter was deleted. Graphs show the percent of cells with each cell cycle outcome (y-axis) upon nutrient addition at different meiotic stages (x-axis). Cells that returned to meiosis are in blue. Cells that were inappropriately uncommitted are in dark grey. Cells that arrested in meiosis I are in green.

We considered that insufficient levels of Ndt80 protein in the P*_GAL1,10_*-*NDT80* strain could cause the inappropriately uncommitted cell phenotype. It has previously been shown that expression from the *GAL1,10* promoter in meiosis does not lead to a large overproduction of the expressed protein [32]. Furthermore, the addition of complete medium with glucose should repress *NDT80* transcription from the *GAL1,1*0 promoter. Therefore, we asked whether Ndt80 protein levels in the *P_GAL1,10_-NDT80* cells decrease with the addition of complete medium. Cells were induced to enter meiosis. When cells arrested at pachytene, estradiol was added to induce the expression of Ndt80. After 110 minutes of Ndt80 induction, complete medium was added and aliquots were taken every 30 minutes for 2.5 hours. The 110-minute timepoint was chosen due to the large fraction of inappropriately uncommitted cells at this timepoint (after 110-minutes of estradiol addition, most of the cells had disassembled their SC 30 minutes earlier, and therefore this timepoint corresponds to the 30-minute timepoint shown in Figure 4B). Analysis of Ndt80 levels by western blot showed that the Ndt80 protein levels decreased by 86% within 30-minutes of complete medium addition and remained at that very low level (Figure 5A, upper panel). In a control experiment, cells that continued in meiosis with estradiol in the sporulation medium maintained the high level of Ndt80 protein (Figure 5A, lower panel).

We next determined if there was a difference between Ndt80 protein levels in the P*_GAL1,10_*-*NDT80* strain with the addition of complete medium compared to a strain with *NDT80* under control of its endogenous promoter. Since the cells with P*_NDT80_*-*NDT80* are not as synchronous as the P*_GAL1,10_*-*NDT80* cells, we added a *cdc20* meiotic null to both strains so that the cells would arrest in metaphase I due to a loss of Cdc20 and remain in meiosis. Cells were induced to enter meiosis. When the P*_GAL1,10_*-*NDT80* cells arrested at pachytene, estradiol was added to induce the expression of *NDT80*. After 110 minutes of *NDT80* induction, complete medium was added and aliquots were taken at 0, 15, and 30 minutes to isolate protein. For the P*_NDT80_*-*NDT80* cells, complete medium was added after 12 hours since the cells were in prometaphase I- metaphase I at this timepoint. The protein was isolated at 0, 15, and 30-minute timepoints after complete medium addition. Analysis of Ndt80 by western blot showed that in the P*_GAL1,10_*-*NDT80* cells, the Ndt80 protein decreased by 92% within 15-minutes of complete medium addition (Figure 5B). In contrast, in the P*_NDT80_*-*NDT80* cells, there was only a 27% decrease of Ndt80 protein 15 minutes and a 55% decrease 30 minutes after the introduction of complete medium. These results suggest that insufficient levels of Ndt80 in P*_GAL1,10_*-*NDT80* cells after complete medium addition is likely to result in the inappropriately uncommitted cells.

Since the levels of Ndt80 decreased with the addition of complete medium in the inducible *NDT80* strain, we predicted that lower levels of Ndt80 could result in an altered commitment point. To test our prediction, we further decreased Ndt80 levels by making a *P_GAL1,10-_NDT80/ndt80Δ* strain. In this strain, one copy of *NDT80* is under the *GAL1,10* promoter and the other copy of *NDT80* is deleted. These cells will progress through meiosis and form spores after the induction of *NDT80* with estradiol. Using our microfluidics assay, we monitored cell cycle outcomes after complete medium addition. In Figure 5C, we compare the cell cycle outcomes of *P_GAL1,10-_NDT80/P_GAL1,10-_NDT80* and *P_GAL1,10-_NDT80/ndt80Δ*. Our results are presented as a graph of the percent of cells with each cell cycle outcome (y-axis) at the different meiotic stages in which complete medium was added (x-axis) (Figure 5C, Supp. Table S5). Our data show an important difference in the percent of committed cells between the different strain backgrounds. In the cells with two copies of *P_GAL1,10-_NDT80*, there are inappropriately uncommitted cells upon nutrient addition in prometaphase I, metaphase I, and anaphase I (n>200 cells counted at each stage). However, most cells were committed to meiosis upon nutrient addition in metaphase I and anaphase I. In contrast, in cells with only one copy of *P_GAL1,10-_NDT80*, most cells did not finish meiosis upon nutrient addition. Of the *P_GAL1,10-_NDT80/ndt80Δ* cells in prometaphase I upon nutrient addition, 98% returned to mitosis (n = 178 cells). Of the cells in metaphase I upon nutrient addition 51% returned to mitosis, 8% finished meiosis, 14% were inappropriately uncommitted, and the remainder arrested in either meiosis I or meiosis II (n = 157 cells). Of the cells in anaphase I upon nutrient addition, 84% were inappropriately uncommitted, 8% arrested in meiosis II, and only 8% finished meiosis (n = 100 cells). These results show that by decreasing the levels of Ndt80, fewer cells commit to finishing meiosis.

### Positive feedback in *NDT80* expression ensures meiotic commitment

A comparison of the two strains, *P_GAL1,10-_NDT80/P_GAL1,10-_NDT80* and *P_GAL1,10-_NDT80/ndt80Δ*, suggests that decreasing the levels of *NDT80* resulted in fewer cells committed to meiosis. Therefore, we predict that there is a threshold level of Ndt80 needed to drive cells through meiotic commitment. In a normal meiosis, the high-level burst of *NDT80* expression at the end of pachytene is driven by an auto-regulatory feedback loop in which Ndt80 enhances its own transcription by binding to MSE sequences found in its own promoter [17], [43]. We hypothesize that positive feedback in *NDT80* induction may ensure that cells reach that switch threshold level of *NDT80* required for meiotic commitment. To test this hypothesis, we eliminated positive feedback by deleting the two 9 bp MSEs in the *NDT80* promoter (*P_NDT80-MSE1Δ,2Δ_-NDT80*). In this strain, *NDT80* can be activated by the Ime1 transcription factor, which binds to an upstream regulatory sequence, but cannot be activated through an Ndt80-dependent autoregulatory loop. We verified by western blot that the *P_NDT80-MSE1Δ,2Δ_-NDT80* cells had a substantially lower level of Ndt80 protein (Figure 5D).

**Figure 6:**
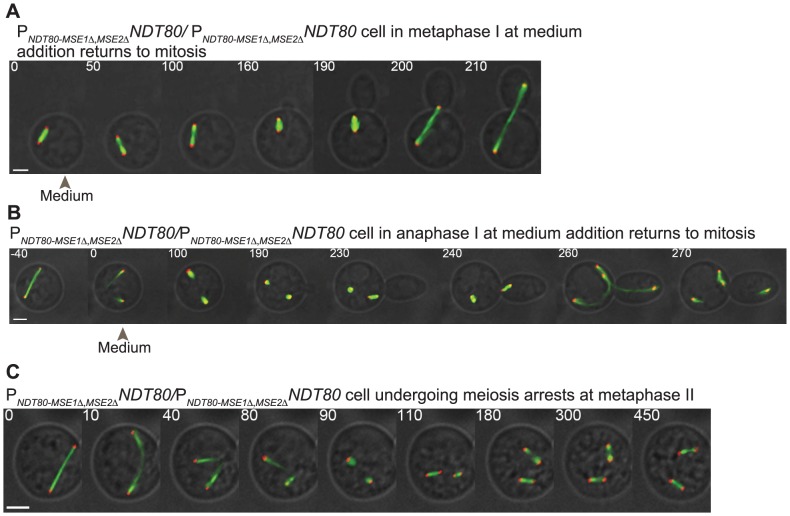
Disruption of positive feedback in *NDT80* expression results in inappropriately uncommitted cells. A-C) Time-lapse images of *P_NDT80-MSE1ΔMSE2Δ_NDT80/P_NDT80-MSE1ΔMSE2Δ_NDT80* cell expressing Spc42-mCherry, Zip1-GFP, and GFP-Tub1 (Scale bar – 3 µm). A) A *P_NDT80-MSE1ΔMSE2Δ_NDT80/P_NDT80-MSE1ΔMSE2Δ_NDT80* cell that buds and returns to mitosis upon complete medium addition (grey arrowhead) in metaphase I. B) A *P_NDT80-MSE1ΔMSE2Δ_NDT80/P_NDT80-MSE1ΔMSE2Δ_NDT80* cell that is inappropriately uncommitted. Complete medium was added (grey arrowhead) to the cell at anaphase I spindle breakdown. Cell finished meiosis I, assembled two spindles, budded, and underwent nuclear division, dividing one nucleus in the mother cell and one nucleus across the bud neck. C) A *P_NDT80-MSE1ΔMSE2Δ_NDT80/P_NDT80-MSE1ΔMSE2Δ_NDT80* cell initiated meiosis and arrested at metaphase II.

We next tested whether the meiotic commitment point was altered in the *P_NDT80-MSE1Δ,2Δ_-NDT80* cells. We performed our microfluidics assay and found that the cells were not committed to meiosis. In Figure 5E, we plot the percent of cells with each cell cycle outcome (y-axis) at the different meiotic stages in which complete medium was added (x-axis)(n = 100 cells counted for each cell-cycle stage) (Supp. Table S6). Of the cells in prometaphase I at the time of complete medium addition, 99% returned to mitosis. *P_NDT80-MSE1Δ,2Δ_-NDT80* cells in metaphase I at the time of nutrient addition gave several cell-cycle outcomes: i) 16% were inappropriately uncommitted and underwent meiosis I, then budded and underwent mitosis, dividing both nuclei; ii) 5% arrested in meiosis I; and iii) 79% budded and returned to mitosis from metaphase I (Video S4, Figure 5E, 6A) (n = 100 cells counted). The *P_NDT80-MSE1Δ,2Δ_-NDT80* cells in anaphase I at the time of complete medium addition were also not committed to meiosis; they finished meiosis I with complete medium addition, but 100% of the cells budded and underwent mitosis instead of meiosis II (Figure 6B, n = 100 cells counted). Cells beyond anaphase I were also inappropriately uncommitted to meiosis; 98% of cells in prophase II at the time of complete medium addition budded and underwent mitosis (n = 100 cells counted). These results show that meiotic commitment requires positive feedback in *NDT80* expression and support our hypothesis that a threshold level of Ndt80 is needed to drive meiotic commitment.

We next asked whether the *P_NDT80-MSE1Δ,2Δ_-NDT80* cells could go through meiosis. We used time-lapse microscopy to monitor the cells undergoing meiosis and found that 78% of the cells that entered meiosis underwent meiosis I but then arrested at metaphase II (Figure 6C, n = 100). Only 5% of the cells finished both meiotic divisions, 10% arrested at pachytene, and 7% arrested in metaphase I. This suggests that the levels of Ndt80 in the *P_NDT80-MSE1Δ,2Δ_-NDT80* strain are sufficient to allow most cells to enter into meiosis I and II, but not sufficient to finish meiosis II. Since the *P_NDT80-MSE1Δ,2Δ_-NDT80* cells do not commit to meiosis, a higher-level induction of *NDT80* is required for meiotic commitment.

### Deleting one copy of *NDT80* delays the meiotic commitment point

**Figure 7:**
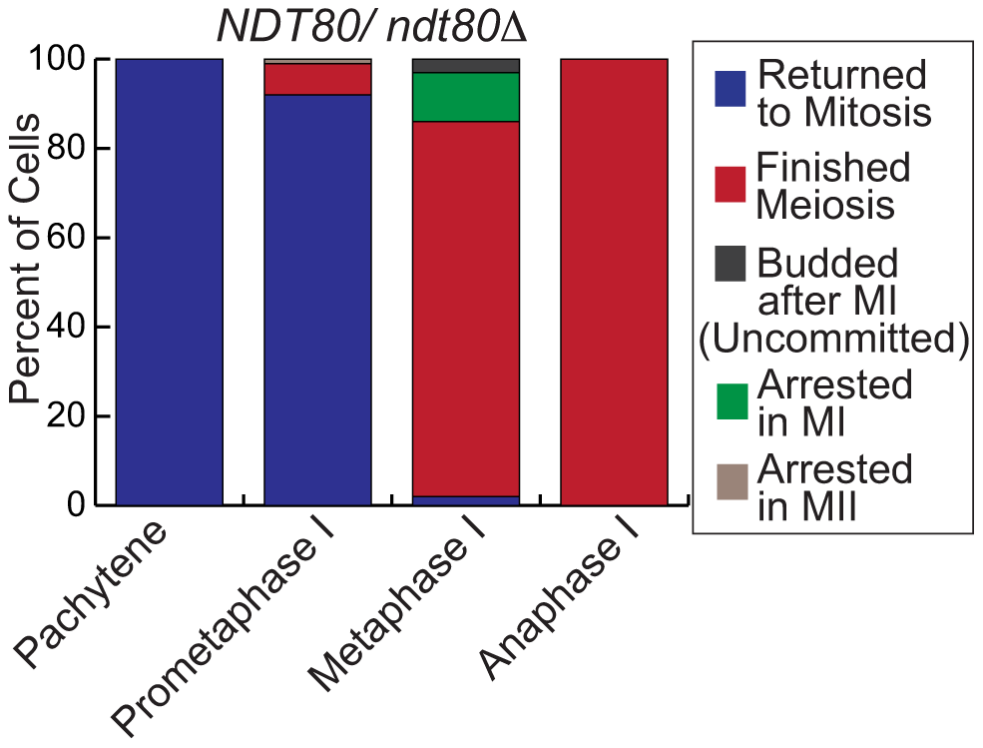
Deletion of one copy of *NDT80* shifts the meiotic commitment point. Graph showing the percent of *NDT80/ndt80Δ* cells at each cell cycle outcome (y-axis) with complete medium addition at the different stages of meiosis (x-axis). Cells that returned to mitosis are in blue. Cells that finished meiosis are in red. Cells that were inappropriately uncommitted are shown in dark grey. Cells that arrest are in green or brown.

To determine if modestly lower levels of *NDT80* result in an alteration of the meiotic commitment point, we decreased Ndt80 levels by making an *NDT80/ndt80Δ* heterozygote. In this strain, one copy of *NDT80* was under the control of its native promoter and the other copy was deleted. Using our microfluidics assay, we found that of the *NDT80/ndt80Δ* cells that were in prometaphase I at complete medium addition, 92% returned to mitosis and 7% finished meiosis (n = 100 cells counted, Figure 7, Supp. Table S3). Of the cells in metaphase I at complete medium addition, 2% returned to mitosis, 84% finished meiosis, 11% arrested in metaphase II, and 3% were inappropriately uncommitted (n = 100 cells counted) (Figure 7). The results show that in *NDT80/ndt80Δ* cells, fewer cells commit to meiosis in prometaphase I when compared to *NDT80/NDT80*, suggesting that the commitment point shifted to the end of prometaphase I/beginning of metaphase I. We conclude that reducing the *NDT80* levels alters the timing of the meiotic commitment point.

Once *NDT80/ndt80Δ* cells exit prophase I, the timing of meiosis was not delayed in comparison to wildtype cells. In wildtype cells, the duration of prometaphase I and metaphase I was 31±1 mins and 28±1 mins, respectively (n = 75 cells counted, average time in minutes ± S.E.). In *NDT80/ndt80Δ* cells, the duration of prometaphase I and metaphase I was 28±1 mins and 21±1 mins, respectively (n = 80 cells, average time in minutes ± S.E.). The timing of the other stages of meiosis were also not delayed (Table 1). These results suggest that although two copies of *NDT80* are needed for the proper timing of meiotic commitment, one copy of *NDT80* is sufficient for the normal duration of each of the meiotic stages.

### Inappropriately uncommitted cells become multi-nucleate

**Figure 8:**
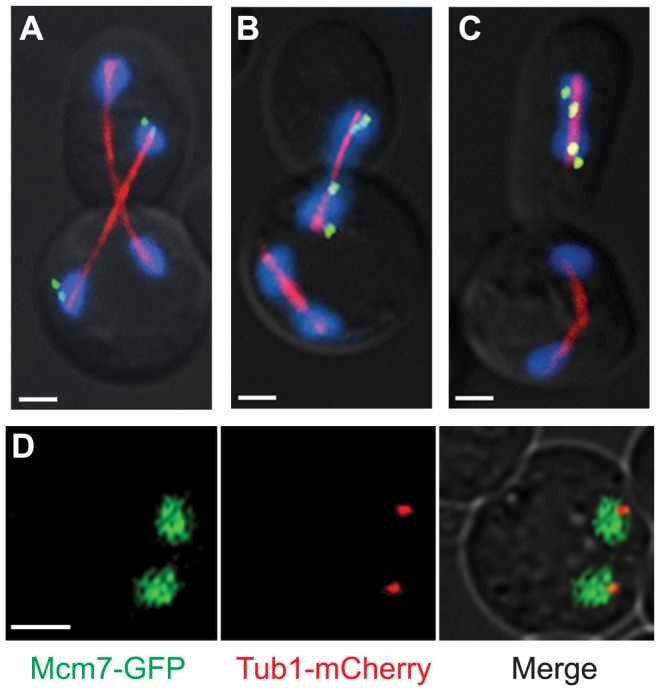
Cells that were inappropriately uncommitted are multinucleate and have an increase in ploidy. A-C) Immunostaining of inappropriately uncommitted cells. The spindle is shown in red with anti-tubulin antibody. The LacO array on chromosome III is marked in green with LacI-GFP and anti-GFP antibody. And, the nucleus is shown in blue with DAPI (Scale bar – 2 µm). There are three nuclear segregation phenotypes. A) Both nuclei divided across the bud neck, resulting in two nuclei in the mother cell and two nuclei in the bud. B) One nucleus divided across the bud neck and one nucleus divided in the mother cell, resulting in three nuclei in the mother cell and one nucleus in the bud. C) One nucleus divided in the mother cell and one nucleus divided in the bud, resulting in two nuclei in the mother cell and two nuclei in the bud. D) Fluorescence microscopy of an inappropriately uncommitted cell that underwent a first division. Mcm7-GFP is shown in green. The spindle is shown in red with mCherry-Tub1 (Scale bar – 3 µm).

The importance of the irreversibility of meiotic commitment can be demonstrated through phenotypic analysis of the inappropriately uncommitted cell; the failure to commit to meiosis beyond prometaphase I results in the formation of multi-nucleate cells. The *P_NDT80-GAL1,10_NDT80/P_NDT80-GAL1,10_NDT80* inappropriately uncommitted cells underwent a first meiotic division, creating two nuclei in the mother cell. After the first meiotic division, the uncommitted cells budded and underwent mitosis instead of finishing meiosis II (Figure 4C). After the mitotic division, depending on how the nuclei divided, the mother cell had either 2 or 3 nuclei and the daughter had either 2 nuclei or 1 nucleus. There were three nuclear segregation phenotypes (Figure 8A-C): i) 36% segregated both nuclei across the bud neck, resulting in two nuclei each in the mother and daughter cells (Figure 8A); ii) 32% segregated one nucleus in the mother cell and one nucleus across the bud neck, resulting in three nuclei in the mother and one in the daughter cell (Figure 8B), and; iii) 32% segregated one nucleus in the mother cell and one nucleus in the daughter cell, resulting in two nuclei each in the mother and daughter cells (Figure 8C) (n = 100). The multi-nucleate mother and daughter cells continued to divide (Video S3) and could increase genome copy number in the subsequent divisions due to the nuclear segregation phenotype described for Figure 8B. Our results show that the loss of meiotic commitment can have the drastic consequence of the loss of genome integrity.

### Inappropriately uncommitted cells replicate their DNA prior to the mitotic division

To determine the ploidy of the inappropriately uncommitted cells, we asked if DNA re-replication occurred after meiosis I but prior to the mitotic division. We monitored the nuclear localization of a component of the replicative helicase, Mcm7, tagged with GFP (Mcm7-GFP). The replicative helicase enters the nucleus and loads onto origins prior to DNA replication [44]. We have previously found that Mcm7-GFP does not enter into the nucleus when cells return to mitosis from prophase I, and these cells also do no re-replicate their DNA [38], [45]. To determine if the inappropriately uncommitted cells license their origins, we monitored the localization of Mcm7-GFP from the strain with *P_NDT80-GAL1,10_NDT80/ndt80Δ*. Using time-lapse microscopy, we found that in the inappropriately uncommitted cells, Mcm7-GFP entered the nuclei after meiosis I but prior to the mitotic division in 92% of the cells (n = 27 cells). Figure 8D shows Mcm7-GFP localized in the two nuclei of an inappropriately uncommitted cell. These results suggest that in the inappropriately uncommitted cells, the levels of CDK decrease after meiosis I such that Mcm7-GFP enters the nuclei and licenses origins. The data support the conclusion that the cell exits meiosis after the first division and then begins the mitotic cell cycle.

To confirm that DNA replication did indeed occur, we monitored a marked chromosome after segregation. We labeled one copy of chromosome III with a lactose operator array (LacO) near the centromere in a strain expressing GFP fused to the lactose repressor (LacI-GFP) [46]. We scored the inappropriately uncommitted cells that were in mitotic anaphase from the *P_GAL1,10_-NDT80/P_GAL1,10_-NDT80* strain. We observed that 74% of the inappropriately uncommitted cells had 3-4 GFP-labeled chromosomes (n = 100, Figure 8A-C), indicating that the cells replicated their DNA prior to the mitotic division. Twenty-six percent had 2 GFP-labeled chromosomes, suggesting that they did not replicate their chromosomes. However, this assay likely under-represents the percent of cells that replicated their chromosomes; due to close attachment of centromeres to the SPB, the GFP labels can often be difficult to resolve [45], [47]. These results indicate that at least 74% of the cells exit meiosis I and begin a mitotic cell cycle in which the cells bud, replicate their DNA (in two separate nuclei), and then segregate their chromosomes. In these cells, there is a 2N DNA content per nucleus after the mitotic division. Therefore, the mother cells with 2-3 nuclei have a 4N-6N DNA content. These results demonstrate that establishing meiotic commitment through the *NDT80* positive feedback loop is important in maintaining ploidy.

## Discussion

The commitment to meiosis has been conceptually defined as the point at which cells can no longer return to mitosis, even in the absence of the inducing environmental signal [9]. However, the molecular mechanisms that regulate this transition are not well understood. We used single-cell analysis to investigate the transcriptional regulatory network that drives the irreversibility of meiotic commitment. We found that high-level induction of *NDT80* is required for the irreversible transition through the meiotic commitment point. Reducing the levels of *NDT80* uncouples meiotic commitment from the entrance into the meiotic division and results in cells that are inappropriately uncommitted to meiosis and can return to mitosis after undergoing anaphase I (Figure 4B,C). Compromising positive feedback of *NDT80* expression, by disrupting the elements that Ndt80 binds within its own promoter, results in a complete loss of commitment; cells return to mitosis from any stage of meiosis (Figure 5E). The return to mitosis after meiosis I is especially detrimental: the mother cell contains two nuclei, buds and then divides both nuclei, creating multi-nucleate mother and daughter cells (Figure 8A-C). Thus, establishing an irreversible meiotic commitment point through positive feedback in *NDT80* expression is essential for genome maintenance.

### Meiotic commitment occurs in prometaphase I

Previous work has shown that upon rich medium addition, cells in pachytene will return to mitosis whereas cells that have entered the meiotic divisions will remain in meiosis [10]–[14]. This led to the prediction that cells commit to meiosis at the end of prophase I as the SPBs begin to separate and meiosis I spindle formation initiates. By monitoring the timing of commitment with respect to the separation of SPBs in individual cells, we were able to show that commitment occurs in prometaphase I, after SPBs have initiated separation, but prior to metaphase I spindle formation. As mentioned by Simchen (2009), the separation of SPBs leads to the establishment of the meiosis I spindle, which may be an important component of commitment. In addition, other possible important components of commitment that occur in prometaphase I could include the expression of the Ndt80-dependent genes, the attachment of homologous chromosomes to spindle microtubules, and the activation of Cdk bound to the M phase cyclins [15], [48], [49]. After these meiotic events occur, the cell will be unable to maintain genome stability in the absence of meiotic commitment. Indeed, we show that inappropriately uncommitted cells that return to mitosis after meiosis I become multi-nucleate with an increase in genome copy number (Figure 8A-C).

### A high-level induction of *NDT80* is required for meiotic commitment

Our results showed that cells become committed to meiosis after the Ndt80-dependent genes are expressed and the encoded proteins are active, including the M phase cyclins Clb1 and Clb4, which initiate meiosis I spindle formation with CDK, and the polo kinase Cdc5, which is needed for SC disassembly [15], [31], [32], [39] (Figure 3). We propose that once cells have entered metaphase I, the levels of proteins, whose expression are dependent on Ndt80, are high enough to drive the cells through the meiotic divisions, even in the absence of the meiosis-inducing signal.

We find that altering *NDT80* expression results in a disruption of commitment. Expression of *NDT80* under the *GAL1,10* promoter resulted in some cells that were inappropriately uncommitted and would return to mitosis after undergoing meiosis I (Figure 4B, C). These cells entered into meiotic divisions but did not commit to meiosis. Western blot analysis shows that the protein levels on Ndt80 drop sharply within 15 minutes of complete medium addition in the P_GAL1,10_-*NDT80/*P_GAL1,10_-*NDT80* cells (Figure 5B). We show that further decreasing the levels of Ndt80, by deleting one of the copies of P_GAL1,10_-*NDT80*, results in a substantial decrease in the percent of cells that commit to finishing meiosis (Figure 5C).

We suggest a model in which there are different threshold levels of Ndt80 required for the entrance into the meiotic divisions and for meiotic commitment: a lower level of Ndt80 promotes meiosis I and meiosis II, but a much higher level is needed to maintain meiosis in the absence of the meiosis-inducing signal and the introduction of the mitosis-inducing signal. Previous work has shown that the RNA levels of *NDT80* and the Ndt80-dependent genes decrease within 30 minutes of nutrient addition, even in committed cells [22]. Therefore, high-level induction of *NDT80* may be needed to obtain a threshold level of protein to sustain meiosis beyond commitment. This suggests that the level of induction of *NDT80* transcription is in excess of what is needed for meiosis, but is required to ensure that the cells can maintain the meiotic pathway once the they have entered into metaphase I.

Besides the genes needed for the meiotic divisions, Ndt80 also transcribes genes whose encoded proteins are required for spore formation [15], [48]. Past work has shown that cells can inappropriately return to mitosis at postmeiotic stages of sporulation [16]. For example, in the absence of *SPO14*, a phospholipase that regulates the formation of the prospore membrane, cells arrest and are able to return to mitosis if rich medium is added [13], [50], [51]. Blocking prospore membrane closure with a mutant of *SSP1* or by temperature upshift also results in the return to mitosis post meiosis [12], [52]. These results suggest that ensuring proper spore formation, possibly through the high-level expression of *NDT80*, is also important to prevent the inappropriate return to mitosis during sporulation.

### Positive feedback in meiotic commitment points

The amplification of a signal through positive feedback can help to make transitions between states irreversible and switch-like. Therefore, positive feedback is an important component of many networks involved in cell-cycle regulation, including those in meiosis [28]. Our work showed that positive feedback of *NDT80* expression has an additional role besides ensuring the switch-like entrance into meiosis: positive feedback ensures the irreversibility of meiotic commitment.

*NDT80* expression is highly regulated through a meiosis-specific transcription factor, a repressor complex that binds to the *NDT80* promoter, and an autoregulatory positive feedback loop [15], [16], [23], [24]. This regulation ensures that *NDT80* is expressed only in cells that are exiting prophase I, and that once it is expressed, a high-level induction ensues. We asked how this regulation affects meiotic commitment. We deleted the transcriptional positive feedback loop by mutating the Ndt80 binding sites within the *NDT80* promoter. In these cells, Ime1 can still induce the expression of *NDT80* but Ndt80 cannot feed back to induce its own expression. In the absence of positive feedback, the cells were inappropriately uncommitted and could return to mitosis from any stage of meiosis. These results show that the high-level induction of *NDT80* through positive feedback is essential for meiotic commitment.

Positive feedback is a common feature in networks that drive irreversible cell-cycle transitions and is also used in the progesterone-dependent commitment to meiotic resumption in the *X. laevis* oocyte. The amphibian oocyte arrests at prophase of meiosis I and the addition of the steroid hormone progesterone releases that arrest by inducing translation of the protein kinase Mos, which activates the mitogen-activated protein kinase cascade (MAPK) and cyclin-dependent kinase (CDK) bound to cyclin B [4], [53]. The cells commit to meiotic maturation 2–3 hours after the addition of progesterone. The cells will remain in the mature state even if only exposed to a transient threshold level of progesterone [54]–[57]. Positive feedback in Mos translation from the activities of Mos, MAPK, and CDK ensures the irreversibility of the commitment to meiotic maturation [54]. When positive feedback is blocked, the response to progesterone becomes transient and reversible. A further study has shown that the threshold response to progesterone can be modulated in response to environmental conditions through linked double-negative feedback loops [58]. In the future, it will be important to determine if nested feedback loops tune the response to the environmental factors that influence meiotic commitment in budding yeast. Comparisons of the network architectures that ensure the irreversibility of the transition through meiotic commitment in different organisms will provide insight into the general properties that govern meiotic commitment points.

## Materials and Methods

### Strains and manipulations

Strains are derivatives of W303 and are listed in Supporting Information Table S1. Deletion and tagged strains were made using standard methods [59]–[61]. Chromosomes were tagged with the *LacO* array and LacI-GFP as described [46]. The inducible *NDT80* strain was made as described [32]. *ZIP1-GFP* with GFP located at the end of the second coiled-coil domain at position 700, replaced *ZIP1* at the endogenous locus as described [35]. To visualize tubulin, constructs containing *P_TUB1_GFP-TUB1* or *P_HIS3_mCherry-TUB1* were integrated into the *URA3* locus. The *NDT80 MSE1Δ MSE2Δ* promoter mutations were made by first cloning *NDT80* with 500 bps of the promoter region to a yeast integrating plasmid (pRS403). The *MSE1* and *MSE2* 9 bp elements were deleted sequentially using a site-directed mutagenesis kit (Finnzymes). Deletions were confirmed by sequencing. The plasmid was integrated into *ndt80*Δ strains at the *HIS3* locus.

### Media

We used the following media: YPD (1% bacto-yeast extract, 2% bacto-peptone, 2% glucose), YPA (1% yeast extract, 2% bacto-peptone, 1% potassium acetate), sporulation medium (1% potassium acetate), and synthetic complete medium (referred to as complete medium, 0.67% bacto-yeast nitrogen base without amino acids, 0.2% dropout mix with all amino acids, 2% glucose).

### Microfluidics assays

In the microfluidics assays, cells were sporulated in liquid culture by growing in YPD at 30°C for 24 hours, diluted into YPA at 30°C for 12–15 h, washed with water and resuspended in sporulation medium at 25°C. Cells were introduced into microfluidics chambers (CellAsic Y04D yeast perfusion plates). Unless otherwise specified, after 12 hours in sporulation medium, synthetic complete medium was flowed into the chambers and cells were monitored by time-lapse microscopy. In the P*_GAL1,10_*-*NDT80* experiments, cells were allowed to reach a prophase arrest in sporulation medium for 11–12.5 hours and then beta-estradiol (1 µM, Sigma) was flowed into the chambers. Synthetic complete medium was then flowed into the chambers at 10-minute timepoints after the addition of beta-estradiol. The RTG assay was performed using the CellAsic Onix microfluidics perfusion platform. The data presented were confirmed in at least three independent experiments.

### Microscope image acquisition and time-lapse microscopy

Cells were imaged using a Nikon Ti-E inverted microscope equipped with a 60× objective (PlanApo n.a.  = 1.4 oil), a Lambda 10-3 optical filter changer and smartshutter (Sutter instrument), GFP and mCherry filters (Chroma Technology), and a CoolSNAPHQ2 CCD camera (Photometrics) at 25°C. Z-stacks of 4–7 sections were acquired in 10 minute intervals for 12–15 h using a 12.5% or 25% ND filter and exposure times of 50–700 ms. Z-stacks were combined into a single maximum intensity projection with NIS-Elements software (Nikon). To measure spindle lengths, 7 Z-stacks were taken at 0.8 µm intervals. The spindle length was measured using the X, Y, and Z with NIS-Elements software.

### Immunofluorescence

For immunofluorescence following the return to mitosis, meiosis-induced cells were cultured in sporulation medium for 10–12 h at 25°C, transferred to synthetic complete medium and incubated for 6.5–7 h at 25°C. Cells were fixed with 4% paraformaldehyde overnight at 4°C, washed twice in phosphate-buffered saline (PBS), resuspended in 1 M sorbitol, and the cell wall was partially digested in 1–1.5 mg/ml Zymolyase (Zymo Research) buffered with 1 M sorbitol for 5–10 minute at room temperature. After washing twice with PBS, cells were immersed in PBS containing 0.2% Triton-X for 10-minutes, then blocked with 5% bovine serum albumin (Sigma) for 1 h at 30°C. Cells were incubated with the primary antibodies, chicken anti-GFP (Molecular Probes, 1∶100), and rabbit anti-Tub1 (1∶250), for 1 h at 30°C. Cells were washed twice in PBS, once in PBS containing 0.1% Tween 20, then once in PBS. The secondary antibodies, Alexa 488 anti-chicken (Molecular Probes, 1∶100) and Alexa 594 anti-rabbit (Molecular Probes, 1∶100) were used. Cells were washed twice in PBS, then stained with 1 µg/ml of DAPI.

### Western blots

For western blots, protein was isolated from 6 mls of cells after complete medium addition. Strains were sporulated in liquid culture by growing in YPD at 30°C to saturation, diluted into YPA for 12–15 h at 30°C, washed with water and resuspended in sporulation medium at 25°C. For the *P_GAL1,10_NDT80* strains, beta-estradiol (1 µM, Sigma) was added 14 hours after the resuspension in potassium acetate. After 110 minutes, cells were washed and synthetic complete medium was added to the cells. For the cells in which *NDT80* was continually expressed, the cells were washed and resuspended in sporulation medium with beta-estradiol. Protein was isolated using the TCA method from cells every 15–30 minutes after the addition of synthetic complete medium for a total of 150 minutes. The following antibodies for western blot analysis were used: anti-Ndt80 (gift of M. Lichten, 1∶10000), anti-Nop1 (Toronto Research Chemicals, 1∶10000). The blots shown (Figure 5A,B, D) were cut in two, with the top half probed with anti-Ndt80 and the bottom half probed with anti-Nop1. The secondary antibody used was a donkey anti-rabbit IgG ECL antibody conjugated to HRP for Ndt80 and goat anti-mouse IgG ECL antibody conjugated to HRP. Protein was detected using Amersham ECL western blotting reagents (GE healthcare).

## Supporting Information

Figure S1 Zip1-GFP is disassembled prior to SPB separation. Cells expressing Zip1-GFP and Spc42-mCherry were monitored by time-lapse fluorescence microscopy. A) Cell showing the timing of Zip1-GFP disassembly and SPB separation. B) Comparison of the timing of SC disassembly to SPB separation in cells expressing Spc42-mCherry, GFP-TUB1, and Zip1-GFP and cells expressing Spc42-mCherry and Zip1-GFP. The duration is the average time (mins) ± S.E.
(EPS)

Table S1 Strains used for this study. All strains are derivatives of W303 (*ade2-1 his3-11,15 leu2-3,112 trp1-1 ura3-1 can1-100*).
(DOCX)

Table S2 Cell-cycle outcome when complete medium is provided to wildtype cells with the following spindle lengths (in µm). Data from Figure 2A.
(DOCX)

Table S3 Cell-cycle outcome of wildtype and *NDT80/ndt80Δ* cells when complete medium is added at different meiotic stages. Data from Figure 2B and Figure 7.
(DOCX)

Table S4 Cell cycle outcome of *P_GAL1,10_-NDT80/P_GAL1,10_-NDT80* cells when complete medium is added at ten-minute timepoints after SC disassembly. Data from Figure 4B.
(DOCX)

Table S5 Cell-cycle outcome of *P_GAL1,10_-NDT80/P_GAL1,10_-NDT80* and *P_GAL1,10_-NDT80/ndt80Δ* cells when complete medium is added at different meiotic stages. Data from Figure 5C.
(DOCX)

Table S6 Cell-Cycle outcome of *P_NDT80-MSE1ΔMSE2Δ_-NDT80/P_NDT80-MSE1ΔMSE2Δ_-NDT80* cells when complete medium is added at different meiotic stages. Data from Figure 5E.
(DOCX)

Video S1 Time-lapse fluorescence microscopy of a cell that returns to mitotic growth from prometaphase I. Budding yeast cell expressing Zip1-GFP, GFP-Tub1, and Spc42-mCherry was induced to enter meiosis in a microfluidic chamber. Complete medium was flowed into the chamber when the cell was at prometaphase I. Cell exits meiosis, buds, and undergoes a mitotic division. Images were taken every 10 minutes for 460 minutes (400 ms/frame).
(MOV)

Video S2 Time-lapse fluorescence microscopy of a cell committed to meiosis. Budding yeast cell expressing Zip1-GFP, GFP-Tub1, and Spc42-mCherry was induced to enter meiosis. Complete medium was flowed into the chamber when the cell was at prometaphase I (based on spindle length). Cell finishes meiosis and forms spores. Images were taken every 10 minutes for 440 minutes (400 ms/frame).
(MOV)

Video S3 Time-lapse fluorescence microscopy of an inappropriately uncommitted cell that undergoes meiosis I, and then forms a bud and undergoes a mitotic division. Budding yeast cell expressing Zip1-GFP and GFP-Tub1 is in metaphase I at the time of complete medium addition. The cell undergoes a division, buds, and then divides one nucleus in the mother cell and one nucleus across the bud neck. The mother cell continues to divide and increases genome copy number until death. Images were taken every 10 minutes for 490 minutes (400 ms/frame).
(MOV)

Video S4 Time-lapse microscopy of a cell that returns to mitotic growth from metaphase I. Budding yeast cell expressing Zip1-GFP, GFP-Tub1, and Spc42-mCherry is in metaphase I at the time of complete medium addition, buds, and then divides. Images were taken every 10 minutes for 420 minutes (400 ms/frame).
(MOV)
