# Supplementary material for: Positive Feedback of NDT80 Expression Ensures Irreversible Meiotic Commitment in Budding Yeast
Source: PLoS Genet. 2014 Jun 5;10(6):e1004398. doi: 10.1371/journal.pgen.1004398 (PMC4046916; doi:10.1371/journal.pgen.1004398)
Supplement: Table S1 — Strains used for this study. All strains are derivatives of W303 (ade2-1 his3-11,15 leu2-3,112 trp1-1 ura3-1 can1-100). (DOCX) [file pgen.1004398.s002.docx]

Supporting Table S1:

| **Strain name** | **Genotype** |
| --- | --- |
| LY1521 | *MATa/α, P_TUB1_-GFP-TUB1:URA3/ ura3-1, ZIP1-GFP (700)/ZIP1* SPC42-mCherry:KAN/ SPC42 |
| LY317 | *MATa/α, ZIP1-GFP (700)/ ZIP1-GFP (700)* SPC42-mCherry:KAN/ SPC42 |
| LY1709 | *MATa/α, PDS1-GFP:HIS3/ PDS1-GFP:HIS3* SPC42-mCherry:KAN/ SPC42 |
| LY1042 | *MATa/α, P_GAL1,10_NDT80:TRP1/ P_GAL1,10_NDT80:TRP1, P_TUB1_-GFP-TUB1:LEU2/ P_TUB1_-GFP-TUB1:LEU2, ZIP1-GFP (700)/ ZIP1, Gal4-ER:URA3/ Gal4-ER:URA3* |
| LY1637 | *MATa/α, P_GAL1,10_NDT80:TRP1/ P_GAL1,10_NDT80:TRP1, P_TUB1_-GFP-TUB1:LEU2/ P_TUB1_-GFP-TUB1:LEU2, Gal4-ER:URA3/ Gal4-ER:URA3, P_CLB2-_CDC20:kanMX/ P_CLB2-_CDC20:kanMX* |
| LY1087 | *MATa/α, P_GAL1,10_NDT80:TRP1/ndt80::kanMX, P_TUB1_-GFP-TUB1:LEU2/leu2-3, ZIP1-GFP (700)/ZIP1, Gal4-ER:URA3/ura3-1* |
| LY274 | *MATa/α, P_HIS3_-mCherry-TUB1:URA3/ P_HIS3_-mCherry-TUB1:URA3, PDS1-GFP:HIS3/ PDS1-GFP:HIS3 P_CLB2-_CDC20:kanMX/ P_CLB2-_CDC20:kanMX* |
| LY1634 | *MATa/α, ndt80::kanMX/ NDT80, P_HIS3_-mCherry-TUB1:URA3/ P_HIS3_-mCherry-TUB1:URA3, PDS1-GFP:HIS3/ PDS1, P_CLB2-_CDC20:kanMX/ P_CLB2-_CDC20:kanMX* |
| LY1573 | *MATa/α, ndt80::kanMX/ ndt80::kanMX, P_TUB1_-GFP-TUB1:URA3/ ura3-1, HIS3* SPC42-mCherry:KAN/ SPC42, *ZIP1-GFP (700)/ ZIP1* |
| LY1682 | *MATa/α, ndt80::kanMX/ ndt80::kanMX, P_NDT80-MSE1ΔMSE2Δ_-NDT80:HIS3/ P_NDT80-MSE1ΔMSE2Δ_-NDT80:HIS3, P_HIS3_-mCherry-TUB1:URA3/ TUB1*, *P_CLB2-_CDC20:kanMX/ P_CLB2-_CDC20:kanMX* |
| LY1215 | *MATa/α, ndt80::kanMX/ ndt80::kanMX, P_NDT80-MSE1ΔMSE2Δ_-NDT80:HIS3/ P_NDT80-MSE1ΔMSE2Δ_-NDT80:HIS3, P_TUB1_-GFP-TUB1-URA3/ura3-1, ZIP1-GFP (700)/ZIP1,* SPC42-mCherry:KAN/SPC42 |
| LY1085 | *MATa/α, NDT80/ndt80::kanMX, P_TUB1_-GFP-TUB1:URA3/ ura3-1, ZIP1-GFP (700)/ ZIP1, LacO:TRP1/trp1-1* |
| LY1742 | *MATa/α, P_GAL1,10_NDT80:TRP1/ ndt80::kanMX, MCM7-GFP:HIS3/ MCM7, P_HIS3_-mCherry-TUB1:LEU2/ P_HIS3_-mCherry-TUB1:LEU2, Gal4-ER:URA3/ ura3-1* |
| LY1082 | *MATa/α, P_GAL1,10_NDT80:TRP1/ P_GAL1,10_NDT80:TRP1, LacO:LEU2/ P_HIS3_-mCherry-TUB1:LEU2, P_HIS3_GFP-LacI:HIS3/his3-11,15, Gal4-ER:URA3/ Gal4-ER:URA3* |
