# Supplementary material for: Positive Feedback of NDT80 Expression Ensures Irreversible Meiotic Commitment in Budding Yeast
Source: PLoS Genet. 2014 Jun 5;10(6):e1004398. doi: 10.1371/journal.pgen.1004398 (PMC4046916; doi:10.1371/journal.pgen.1004398)
Supplement: Table S2 — Cell-cycle outcome when complete medium is provided to wildtype cells with the following spindle lengths (in µm). Data from Figure 2A. (DOCX) [file pgen.1004398.s003.docx]

Supporting Table S2.

| Spindle Length (μm) | Cell-cycle outcome |
| --- | --- |
| 1.0 – 1.49 | 100% Return to Mitosis |
| 1.5 - 1.99 | 100% Return to Mitosis |
| 2.0 – 2.49 | 59% Return to Mitosis  41% Finish Meiosis |
| 2.5 – 2.99 | 15% Return to Mitosis  84% Finish Meiosis  1% Arrest in Meiosis I |
| 3.0 – 3.49 | 99% Finish Meiosis  1% Arrest in Meiosis I |
| 3.5 – 3.99 | 99% Finish Meiosis  1% Arrest in Meiosis I |
| 4.0 – 4.5 | 100% Finish Meiosis |
