# Supplementary material for: Positive Feedback of NDT80 Expression Ensures Irreversible Meiotic Commitment in Budding Yeast
Source: PLoS Genet. 2014 Jun 5;10(6):e1004398. doi: 10.1371/journal.pgen.1004398 (PMC4046916; doi:10.1371/journal.pgen.1004398)
Supplement: Table S3 — Cell-cycle outcome of wildtype and NDT80/ndt80Δ cells when complete medium is added at different meiotic stages. Data from Figure 2B and Figure 7. (DOCX) [file pgen.1004398.s004.docx]

**Supporting Table S3.**

| Meiotic stage at complete medium addition | Wildtype  Cell-cycle outcome | *NDT80/ndt80∆* |
| --- | --- | --- |
| Pachytene | 100% Returned to Mitosis | 100% Returned to Mitosis |
| Prometaphase I | 64% Returned to Mitosis  34% Finished Meiosis  2% Arrested in Meiosis I | 92% Returned to Mitosis  7% Finished Meiosis  1% Arrested in Meiosis II |
| Metaphase I | 99% Finished Meiosis  1% Arrested in Meiosis I | 2% Returned to Mitosis  84% Finished Meiosis  3% Budded after Meiosis I  11% Arrested in Meiosis I |
| Anaphase I | 100% Finished Meiosis | 100% Finished Meiosis |
