# Supplementary material for: Positive Feedback of NDT80 Expression Ensures Irreversible Meiotic Commitment in Budding Yeast
Source: PLoS Genet. 2014 Jun 5;10(6):e1004398. doi: 10.1371/journal.pgen.1004398 (PMC4046916; doi:10.1371/journal.pgen.1004398)
Supplement: Table S4 — Cell cycle outcome of PGAL1,10-NDT80/PGAL1,10-NDT80 cells when complete medium is added at ten-minute timepoints after SC disassembly. Data from Figure 4B. (DOCX) [file pgen.1004398.s005.docx]

Supporting Table S4**:**

| Time of Complete Medium Addition after SC Disassembly (mins) | *P_GAL1,10_-NDT80/ P_GAL1,10_-NDT80* Cell-cycle outcome |
| --- | --- |
| 0 | 100% Returned to Mitosis |
| 10 | 99% Returned to Mitosis  1% Arrested in Meiosis I |
| 20 | 42% Returned to Mitosis  19% Finished Meiosis  36% Budded after Meiosis I  1% Arrested after Meiosis I  2% Arrested after Meiosis II |
| 30 | 5% Returned to Mitosis  60% Finished Meiosis  32% Budded after Meiosis I  1% Arrested after Meiosis I  2% Arrested after Meiosis II |
| 40 | 1% Returned to Mitosis  93% Finished Meiosis  4% Budded after Meiosis I  2% Arrested after Meiosis I |
| 50 | 100% Finished Meiosis |
| 60 | 100% Finished Meiosis |
| 70 | 100% Finished Meiosis |
| 80 | 100% Finished Meiosis |
