# Supplementary material for: Positive Feedback of NDT80 Expression Ensures Irreversible Meiotic Commitment in Budding Yeast
Source: PLoS Genet. 2014 Jun 5;10(6):e1004398. doi: 10.1371/journal.pgen.1004398 (PMC4046916; doi:10.1371/journal.pgen.1004398)
Supplement: Table S5 — Cell-cycle outcome of PGAL1,10-NDT80/PGAL1,10-NDT80 and PGAL1,10-NDT80/ndt80Δ cells when complete medium is added at different meiotic stages. Data from Figure 5C. (DOCX) [file pgen.1004398.s006.docx]

Supporting Table S5.

| Meiotic stage at complete medium addition | *P_GAL1,10_-NDT80/ P_GAL1,10_-NDT80* | *P_GAL1,10_-NDT80/ ndt80∆* |
| --- | --- | --- |
| Pachytene | 100% Returned to Mitosis | 99% Returned to Mitosis  1% Arrested in Meiosis I |
| Prometaphase I | 63% Returned to Mitosis  9% Finished Meiosis  23% Budded after Meiosis I  1% Arrested in Meiosis I  4% Arrested in Meiosis II | 98% Returned to Mitosis  2% Arrested in Meiosis I |
| Metaphase I | 64% Finish meiosis  33% Budded after Meiosis I  1% Arrested in Meiosis I  2% Arrested in Meiosis II | 51% Returned to Mitosis  8% Finished Meiosis  14% Budded after Meiosis I  11% Arrested in Meiosis I  16% Arrested in Meiosis II |
| Anaphase I | 85% finish meiosis  13% Budded after Meiosis I  1% Arrested in Meiosis I  1% Arrested in Meiosis II | 8% Finished Meiosis  84% Budded after Meiosis I  8% Arrested in Meiosis II |
