# Supplementary material for: Positive Feedback of NDT80 Expression Ensures Irreversible Meiotic Commitment in Budding Yeast
Source: PLoS Genet. 2014 Jun 5;10(6):e1004398. doi: 10.1371/journal.pgen.1004398 (PMC4046916; doi:10.1371/journal.pgen.1004398)
Supplement: Table S6 — Cell-Cycle outcome of PNDT80-MSE1ΔMSE2Δ-NDT80/PNDT80-MSE1ΔMSE2Δ-NDT80 cells when complete medium is added at different meiotic stages. Data from Figure 5E. (DOCX) [file pgen.1004398.s007.docx]

Supporting Table S6**:**

| Meiotic stage at complete medium addition | *P_NDT80-MSE1∆MSE2∆_-NDT80/ P_NDT80-MSE1∆MSE2∆_-NDT80* |
| --- | --- |
| Pachytene | 100% Returned to Mitosis |
| Prometaphase I | 99% Returned to Mitosis  1% Budded after Meiosis I |
| Metaphase I | 82% Finished Meiosis  13% Budded after Meiosis I  5% Arrested in Meiosis I |
| Anaphase I | 100% Budded after Meiosis I |
